# CMR T1 and T2 mapping and extracellular volume quantification in systolic phase produces superior image quality with less motion artifacts and equal mapping values compared to conventional diastolic mapping

**DOI:** 10.1007/s10334-026-01353-8

**Published:** 2026-04-15

**Authors:** Johanna Federico, Suvi Syväranta, Miia Holmström, Jyri Lommi, Satu Suihko, Ritva Peltomaa, Riitta Koivuniemi, Marjatta Leirisalo-Repo, Juha Peltonen, Touko Kaasalainen, Satu Vaara

**Affiliations:** 1 Radiology, HUS Diagnostic Center, University of Helsinki and Helsinki University Hospital, Haartmaninkatu 4 Rakennus 1, 00290 Helsinki, Finland; 2 Cardiology, HUS Heart and Lung Center, University of Helsinki and Helsinki University Hospital, Haartmaninkatu 4 Rakennus 1, 00290 Helsinki, Finland; 3 Rheumatology, University of Helsinki and Helsinki University Hospital, Haartmaninkatu 4 Rakennus 1, 00290 Helsinki, Finland

**Keywords:** CMR, Systolic mapping, Imaging artifacts

## Abstract

**Aim:**

This study investigated whether systolic phase imaging improves cardiac magnetic resonance (CMR) mapping by reducing artifacts compared with diastolic phase analyses. We evaluated T1 and T2 relaxation times and extracellular volume (ECV) in healthy individuals, early rheumatoid arthritis (ERA), and aortic stenosis (AS) patients.

**Materials and methods:**

A total of 114 participants (54 healthy adults, 30 ERA and 30 AS patients) underwent 1.5 T CMR with T1, T2, and ECV mapping in both diastolic and systolic phases. Imaging artifacts were categorized as minor or major, and inter-observer agreement was assessed for image quality and reproducibility.

**Results:**

Systolic phase mapping significantly reduced minor imaging artifacts across all cohorts (14% vs 5%, *p* < 0.001 for T1; 5% vs 2%, *p* < 0.001 for T2 in healthy cohort), particularly in participants with thinner myocardium. Inter-observer agreement assessed in the ERA cohort was excellent, with higher intraclass correlation coefficients (ICC) for systolic than diastolic mapping, particularly for T2 (0.975 vs 0.867) and ECV (0.951 vs 0.891). Systolic mapping yielded comparable T1 and T2 relaxation times across all cohorts.

**Conclusions:**

Systolic mapping provides comparable T1, T2, and ECV values to diastolic mapping, while reducing artifacts, supporting its clinical applicability across diverse patient profiles.

**Supplementary Information:**

The online version contains supplementary material available at https://doi.org/10.1007/s10334-026-01353-8.

## Introduction

Cardiac magnetic resonance (CMR) imaging is essential for diagnosing and managing cardiovascular diseases [1, 2]. T1 and T2 mapping sequences, combined with extracellular volume quantification (ECV), enable precise myocardial tissue characterization, with T1 and T2 mapping offering gadolinium-free assessment [2–5]. These techniques are used to evaluate cardiac pathologies, such as myocardial fibrosis, infarction, cardiomyopathies, and myocarditis [1, 2, 6–15].

T1, T2, and ECV mapping are sensitive to artifacts, with even minor variations in CMR field strength affecting results [2, 5, 16]. The T1-mapping sequences MOLLI and ShMOLLI demonstrate excellent inter- and intraobserver reproducibility [2, 17]. T1- and T2-mapping sequences are traditionally acquired in diastolic phase to minimize motion artifacts [5]. Endo- and epicardial borders are contoured to exclude adjacent tissues and artifacts caused by suboptimal slice positioning, failed breath holds, mistriggering, or unexpected tachyarrhythmias [18, 19]. The partial-volume effect is a significant source of artifacts in mapping, when a voxel at the myocardium–blood pool border contains blood signal intensity, resulting in measurement inaccuracies, especially in individuals with thin myocardial walls [9, 18].

Previous research shows good agreement between systolic and diastolic phase mapping in patients with atrial fibrillation, while reducing artifacts in systolic mapping [20]. Research on healthy volunteers suggests clinically equivalent T1 values between diastolic and systolic phase mapping, but the study cohorts have been small and T1 relaxation times have shown statistically significant differences, likely due to reduction in partial-volume effect in systolic mapping [18, 21]. Our aim is to investigate whether T1, T2, and ECV mapping acquisitions in systolic phase produce comparable mapping results to traditional diastolic acquisitions, with significantly fewer motion artifacts, in healthy adult volunteer population and in patients with treatment naïve early rheumatoid arthritis (ERA) and aortic valve stenosis (AS).

## Methods

### Study population

This study recruited 60 self-reportedly healthy adult volunteers with an in-house Helsinki University Hospital intranet advertisement. Participants from various occupational groups were included, excluding those individuals with any previous heart condition (such as diagnosed hypertension, hypercholesterolemia, and coronary artery disease), asthma, diabetes mellitus, any autoimmune disease, and self-reported cardiovascular symptoms, pregnancy, or any other CMR contraindication. Participants completed a medical history survey and provided written informed consent. The study was approved by the hospital research committee (HUS/31/2024) and the local ethics committee (HUS/2471/2019), and the Declaration of Helsinki was followed.

Additionally, we enrolled 30 randomly selected, recently diagnosed patients with early rheumatoid arthritis (ERA, ethics committee decision; HUS/26/2018 and research number HUS/2691/2018) and 30 patients with aortic stenosis (AS, research number HUS/46/2018; ethics committee decision HUS/1743/2018) from other studies into this research [22, 23]. These patient groups were selected to evaluate mapping quantities across varying myocardial wall thicknesses. AS patients commonly exhibit ventricular hypertrophy due to chronic systolic pressure overload [24], whereas previous studies have shown that ERA patients tend to have reduced myocardial mass compared to healthy individuals [25, 26].

Six of the healthy volunteers were excluded due to unexpected CMR findings; one volunteer had a severely reduced ejection fraction (EF) and myocardial strain, one was diagnosed with asymptomatic mitral valve regurgitation, one had moderate-to-severe coronary artery disease, and three showed septal myocardial thickening suggestive of undiagnosed hypertension. These findings were assessed by a consulting cardiologist, and the patients involved were notified and referred to a medical professional. One patient was diagnosed with clinical hypertension afterward.

### CMR scan protocol

All participants underwent CMR using a 1.5 T scanner (Siemens Avanto Fit, Erlangen, Germany) at Meilahti University Hospital. A 30-channel body coil and 32-channel spine coil were used. The healthy control group underwent a non-contrast CMR due to ethical considerations, while ERA and AS patients also had hematocrit-based ECV analyses.

To minimize respiratory motion artifacts, breath-hold acquisitions were performed. Retrospectively ECG-gated balanced steady-state free precession (bSSFP) cine sequences were used to evaluate LV and RV volumes and EF in vertical, horizontal, and short-axis views. Key cine imaging parameters included: TR 2.8 ms, TE 1.2 ms, flip angle 55°, matrix size 192 × 154, FOV 280 mm × 230 mm, slice thickness 6 mm, inter-slice gaps of 20–30%, and 30 reconstructed cardiac phases. Temporal resolution ranged from 34 to 40 ms.

T2-short tau inversion-recovery images were acquired at the apical, mid-ventricular, and basal LV levels. Key parameters: TR of two heartbeats, TE 45 ms, matrix 192 × 148, FOV 270 × 245 mm, slice thickness 8 mm. T2 mapping used a T2prep SSFP sequence capturing images at three T2 times (0, 25, and 55 ms) in diastolic and systolic phases.

Pre- and post-contrast T1 mapping used the shortened Modified Look-Locker Inversion-recovery (shMOLLI) sequence in short-axis views. Parameters: TR/TE 2.4/1.2 ms, flip angle 35°, matrix 256 × 169, FOV 300 × 256 mm, and slice thickness 8 mm. Post-contrast imaging was performed 12 min after administering gadoterate meglumine (Dotarem^®^ 0.2 mmol/kg).

All parametric mapping acquisitions (T1 and T2 mapping) were performed using prospective ECG-triggering. Diastolic phase mapping was defined by the scanner as the latest available acquisition window before the subsequent R-wave, corresponding to end-diastole. Systolic phase mapping was prescribed using a fixed trigger delay of 100 ms after the R-wave. Heart-rate adaptation was handled automatically by the scanner based on the preceding RR interval. The nominal acquisition window for parametric mapping was approximately 350 ms, including preparation pulses.

Late gadolinium enhancement (LGE) imaging used a PSIR sequence with TR/TE 2.5/1.1 ms, inversion time 350–400 ms, flip angle 40°, and FOV 270 × 200 mm.

### Image post-processing

**Fig. 1:**
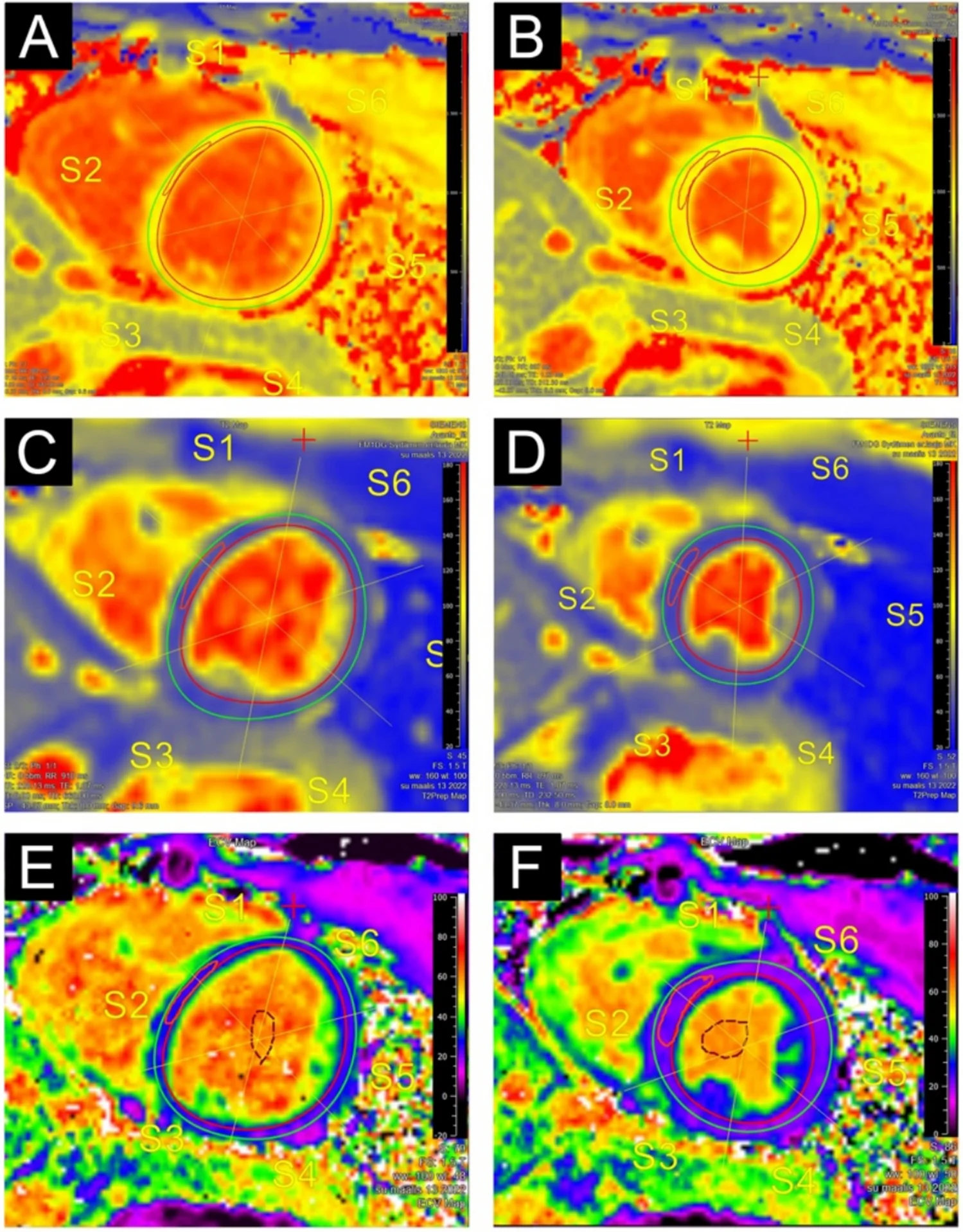
T1 and T2 mapping and extracellular volume (ECV) mapping analyses in diastolic and systolic phases using Medis software. Endocardial and epicardial contours and a region of interest (ROI) are drawn to each slice. Pictures **A, B** show T1, **C, D** T2, and **E, F** extracellular volume (ECV) mid-septal mapping images with ROI drawn in red color in the mid myocardial wall. Diastolic phase images are on the left and systolic on the right

DICOM images were processed using QMass analysis software (Medis Suite v4.0.24.4, Medis Medical Imaging Systems, The Netherlands). T1 and T2 mapping source images were motion-corrected using the vendor´s algorithm. The myocardium was delineated in basal, mid, and apical planes, by drawing endocardial and epicardial contours in diastole and systole, excluding voxels with blood and epicardial fat (Fig. 1). The myocardium was divided into 16 segments per American Heart Association (AHA) guidelines, excluding the apical cap (segment 17), and segmental analyses for T1, T2, and ECV were performed. Global values were calculated as the mean of all segments.

The mid-ventricular septum region is considered less susceptible to motion-related artifacts and partial-volume effects [5]. Therefore, a region of interest (ROI) was manually drawn according to international guidelines between the segments 8 and 9 in both diastole and systole to reduce the impact of segmental artifacts and to improve mapping reproducibility.

An experienced cardiac radiologist (S.S.), with over 5 years of CMR experience, analyzed all the images and marked segments with artifacts. The analysis was designed to ensure that even subtle artifacts, which might be overlooked in routine clinical workflows, were taken into account. Prior to categorization, additional manual translation was applied to pre- and post-T1 maps for the analysis if residual misalignment was observed after vendor-provided motion correction, including both intra-sequence motion and inter-sequence motion in ECV calculation. The categorization was performed following clinical image interpretation. Artifacts, mainly motion artifacts or banding, were categorized as minor if the segment remained at least partially analyzable, and major if the whole segment was clinically considered unreliable and had to be excluded (Supplementary Fig. 1). To avoid exaggerating the benefits of systolic mapping, artifacts resulting from suboptimal basal slice positioning were included in the analyses. To assess the inter-observer agreement between two experienced cardiac radiologists (S.S. and S.V.) reading the CMR scans, the intraclass correlation coefficient was calculated from the ERA cohorts global mapping results (derived from segmental mapping results), reporting the average measures intraclass correlation (ICC). The observers were blinded for the detailed clinical data.

### Statistical analysis

All statistical analyses were performed using IBM SPSS Statistics (version 28.0, IBM SPSS Statistics, Corp., Armonk, New York, USA). Due to a relatively small sample size, nonparametric tests were used, and results are given as medians with interquartile range (IQR [Q1, Q3]). The related-samples Wilcoxon signed-rank test was used to compare relaxation times and ECV quantification, while the Kruskal–Wallis test assessed group differences. For artifact analysis, minor and major artifacts were analyzed separately as dichotomous variables (presence vs absence), and paired comparisons between diastolic and systolic phase mapping were performed using the McNemar test. Bland–Altman plots, Spearman’s correlation, and linear regression evaluated agreement between diastolic and systolic mapping and detected systematic bias. All statistical analyses were two-sided, and a *p* value ≤ 0.05 was considered statistically significant.

## Results

The analysis was conducted with 54 healthy volunteers, 30 AS cohort patients, and 30 ERA cohort patients, a total of 114 subjects. The baseline characteristics of cohorts are displayed in Table 1. The median age was 45.9 [32.8–58.9] years in the healthy cohort, 61.9 [47.7–67.4] years in the ERA group, and 76.4 [70.4–82.3] years in the AS group. LV myocardial mass index was 50.0 [44.9–57.5] g/m^2^ in the control group, 47.2 [40.3–52.3] g/m^2^ in the ERA group, and 56.1 [52.2–65.7] g/m^2^ in the AS group; the AS group demonstrating significantly higher LV mass than the two groups indicating myocardial hypertrophy (*p* = 0.007 for healthy cohort vs AS cohort and *p* < 0.001 for ERA cohort vs AS cohort).

**Table 1:** Baseline characteristics of the study cohorts

	Healthy cohort	ERA group	AS group
Number of subjects	54	30	30
Age (years)	45.9 [32.8–58.9]	61.9 [47.7–67.4]	76.4 [70.4–82.3]
Sex (women), number (%)	27 (50.0)	23 (76.6)	8 (26.7)
BMI (kg/m^2^)	24.9 [22.6–27.5]	26.6 [23.7–29.2]	26.3 [24.3–27.4]
BSA (m^2^)	1.94 [1.80–2.01]	1.88 [1.66–2.04]	1.94 [1.82–2.03]
Heart rate (bpm)	64.4 [61.0–74.5]	73.5 [65.8–83.0]	67.0 [59.8–76.5]
LV EDV index (ml/m^2^)	82.7 [72.9–95.8]	76.5 [67.8–84.3]	70.9 [62.3–76.2]
LV ESV index (ml/m^2^)	32.5 [26.8–40.6]	29.2 [25.0–33.1]	25.4 [21.8–29.16]
LV EF (%)	60.8 [58.1–63.5]	61.8 [58.1–66.7]	62.4 [57.8–68.5]
LV mass index (g/ m^2)^	50.0 [44.9–57.5]	47.2 [40.3–52.3]	56.1 [52.2–65.7]
RV EDV index (ml/m^2^)	87.2 [75.3–98.2]	75.6 [64.6–83.2]	65.6 [58.2–73.9]
RV ESV index (ml/m^2^)	36.3 [26.9–44.1]	27.6 [20.9–32.6]	25.4 [19.8–30.2]
RV EF (%)	59.1 [55.1–62.8]	64.0 [59.1–67.9]	61.9 [58.0–67.2]

The results are medians with interquartile ranges [IQR1–IQR3].

*ERA* early rheumatoid arthritis, *AS* aortic stenosis, *BMI* body mass index, *BSA* body surface area, *bpm* beats per minute, *LV EDV* left ventricle end-diastolic volume, *LV ESV* end-systolic volume, *LV EF* left ventricle ejection fraction, *LA* left atrium, *RV EDV* right ventricle end-diastolic volume, *RV ESV* right ventricle end-systolic volume, *RV EF* right ventricle ejection fraction

### Agreement between analysts

The ICC was calculated from the mapping results of the ERA cohort (*n* = 30), as their thin myocardial walls and susceptibility to partial-volume effect make their mapping results the most challenging to interpret. The agreement between the two experienced cardiac imaging radiologists was excellent across all parameters (Table 2). Notably, the ICC for systolic mapping was slightly higher compared to diastolic mapping in all categories. The improvement was greatest in T2 mapping (0.867 vs 0.975).

**Table 2:** The intraclass correlation (ICC) for the global T1, T2, and extracellular volume (ECV) mapping results in early rheumatoid arthritis (ERA) cohort

	Diastolic mapping	Systolic mapping
T1	0.976	0.979
T2	0.867	0.975
ECV	0.891	0.951

*ECV* extracellular volume

### T1 mapping

The mid-septal ROI T1 relaxation times showed comparable results between diastolic and systolic mapping across all study cohorts (in the healthy cohort 972 [958–999] ms vs 972 [953–998] ms, *p* = 0.583; in the ERA cohort 957 [939–980] ms vs 963 [938–984] ms, *p* = 0.766; in the AS cohort 977 [957–995] ms vs 984 [953–995] ms, *p* = 0.491). More detailed mapping results are presented in Table 3.

**Table 3:** The mid-septal ROI mapping results across all study cohorts

	Diastolic phase	Systolic phase	*p* value
Healthy cohort			
T1 (ms)	972 [958–999]	972 [953–998]	0.583
T2 (ms)	45.0 [43.0–47.0]	44.5 [43.0–46.0]	0.173
ERA group			
T1 (ms)	957 [939–980]	963 [938–984]	0.766
T2 (ms)	45.5 [43.9–47.4]	44.5 [43.2–46.0]	0.141
ECV (%)	25.8 [24.6–27.4]	24.1 [22.7–26.3]	0.003
AS group			
T1 (ms)	977 [957–995]	984 [953–995]	0.491
T2 (ms)	46.0 [44.4–47.9]	45.6 [44.5–47.6]	0.785
ECV (%)	26.7 [23.0–28.4]	26.5 [24.3–28.2]	0.336

The results are medians with interquartile ranges [IQR1–IQR3]

*ERA* early rheumatoid arthritis, *ECV* extracellular volume, *AS* aortic stenosis, *ROI* region of interest

**Fig. 2:**
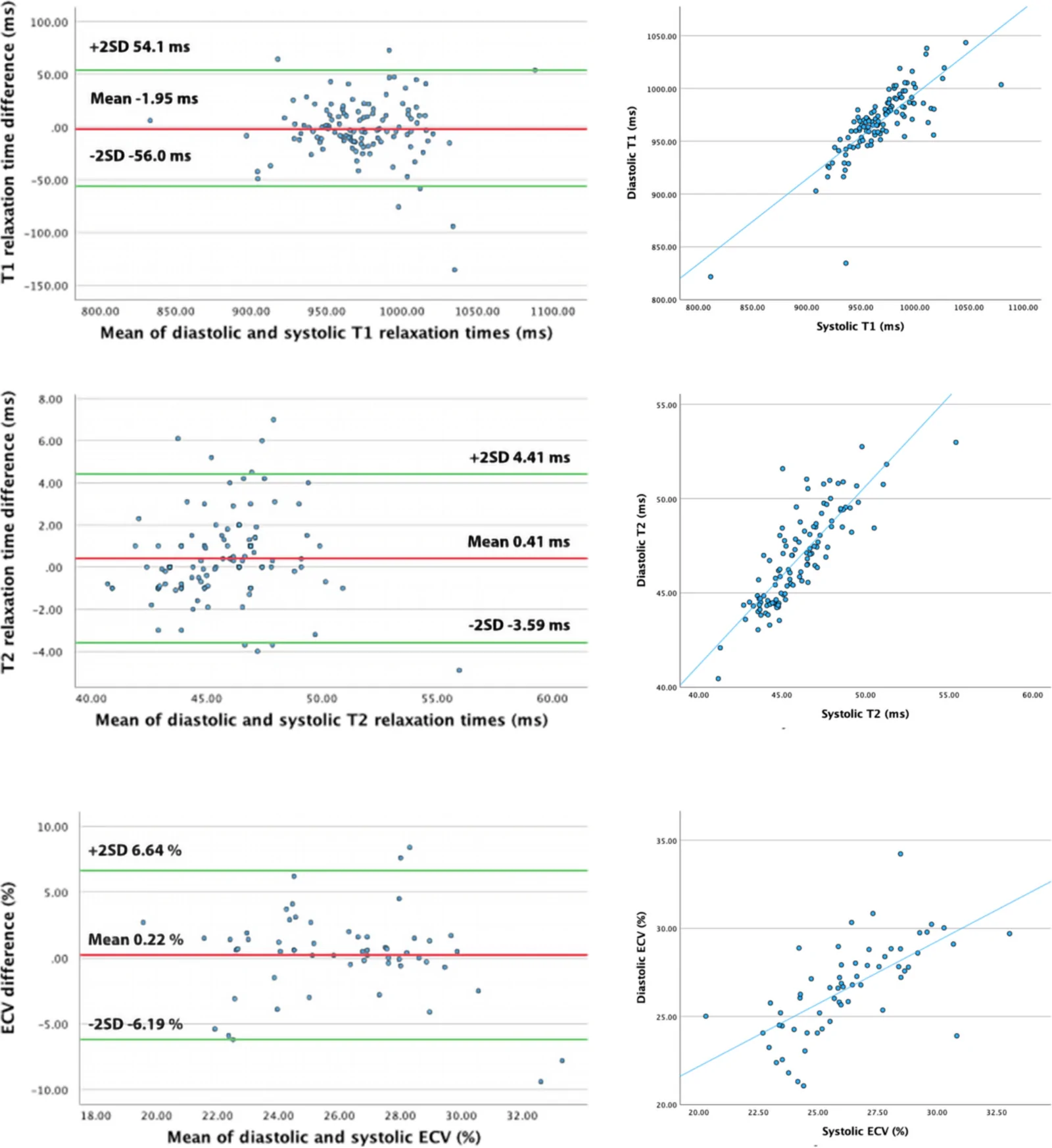
The Bland–Altman plots (left) and scatter plots (right) of all study cohorts comparing systolic and diastolic global T1 and T2 relaxation times and extracellular volume (ECV) values. Linear regression lines are overlaid on the scatter plots to illustrate potential systematic trends. Correlation strength was quantified using Spearman’s correlation, as reported in the Results section. *ECV* extracellular volume; *SD* standard deviation

The Bland–Altman plots (Fig. 2) were created for T1 with the combined relaxation times of all patient cohorts, and showed high agreement (bias – 1.95 ms and limits of agreement ± 56 ms, *p* = 0.469). The Spearman’s correlation analysis revealed a strong positive correlation for T1 systolic and diastolic mapping (*r* = 0.766, *p* < 0.001).

### T2 mapping

The mid-septal ROI T2 relaxation times were similar in diastolic and systolic mapping across all study cohorts (Table 3). The Bland–Altman plots highlighted high agreement for T2 relaxation times (bias 0.86 ms and limits of agreement ± 2.67 ms, *p* = 0.035) and the Spearman’s correlation analysis demonstrated a good positive correlation (*r* = 0.675, *p* < 0.001).

### ECV quantification

The ECV quantification was performed from the contrast CMR studies in the ERA and AS groups. In the AS group, mid-septal ECV values were comparable between diastolic and systolic mapping (26.7 [23.0–28.4] % vs 26.5 [24.3–28.2] %, *p* = 0.336). In the ERA group, a small but statistically significant reduction in ECV was noted (25.8 [24.6–27.4] % vs 24.1 [22.7–26.3] %, *p* = 0.003). In the Spearman’s analysis, a good positive correlation was found between ECV measurements in diastolic and systolic mapping (*r* = 0.634, *p* < 0.001), and the Bland–Altman plots demonstrated good agreement between the two methods (bias 0.2%, limits of agreement ± 6.4%, *p* = 0.606) (Fig. 2).

### Global and segmental mapping

The global T1 and T2 relaxation times measured during the diastolic and systolic phases were statistically different across all study cohorts. In the healthy cohort, T1 values measured 971 [960–990] ms in the diastolic phase vs 967 [953–986] ms in the systolic phase (*p* = 0.007), and T2 values 45.9 [44.5–48.5] ms vs 45.5 [44.1–47.0] ms (*p* < 0.001). In the ERA group, T1 values measured 969 [950–980] ms vs 960 [943–976] ms (*p* = 0.006), and T2 values 47.0 [45.0–48.1] ms vs 45.2 [44.7–46.7] ms (*p* < 0.001). In the AS group, T1 values were 961 [949–985] ms vs 975 [949–998] ms (*p* = 0.010), and T2 values 47.4 [44.8–49.8] ms vs 46.5 [45.1–47.8] ms (*p* = 0.014). Global ECV quantification also showed differences in the ERA cohort, with values of 26.5 [24.9–28.5] % vs 25.5 [23.9–26.7] % (*p* < 0.001), while in the AS cohort, ECV mapping values were similar between diastolic and systolic phase mapping (27.0 [24.4–28.7] % vs 26.7 [25.4–28.9] %, *p* = 0.079).

**Fig. 3:**
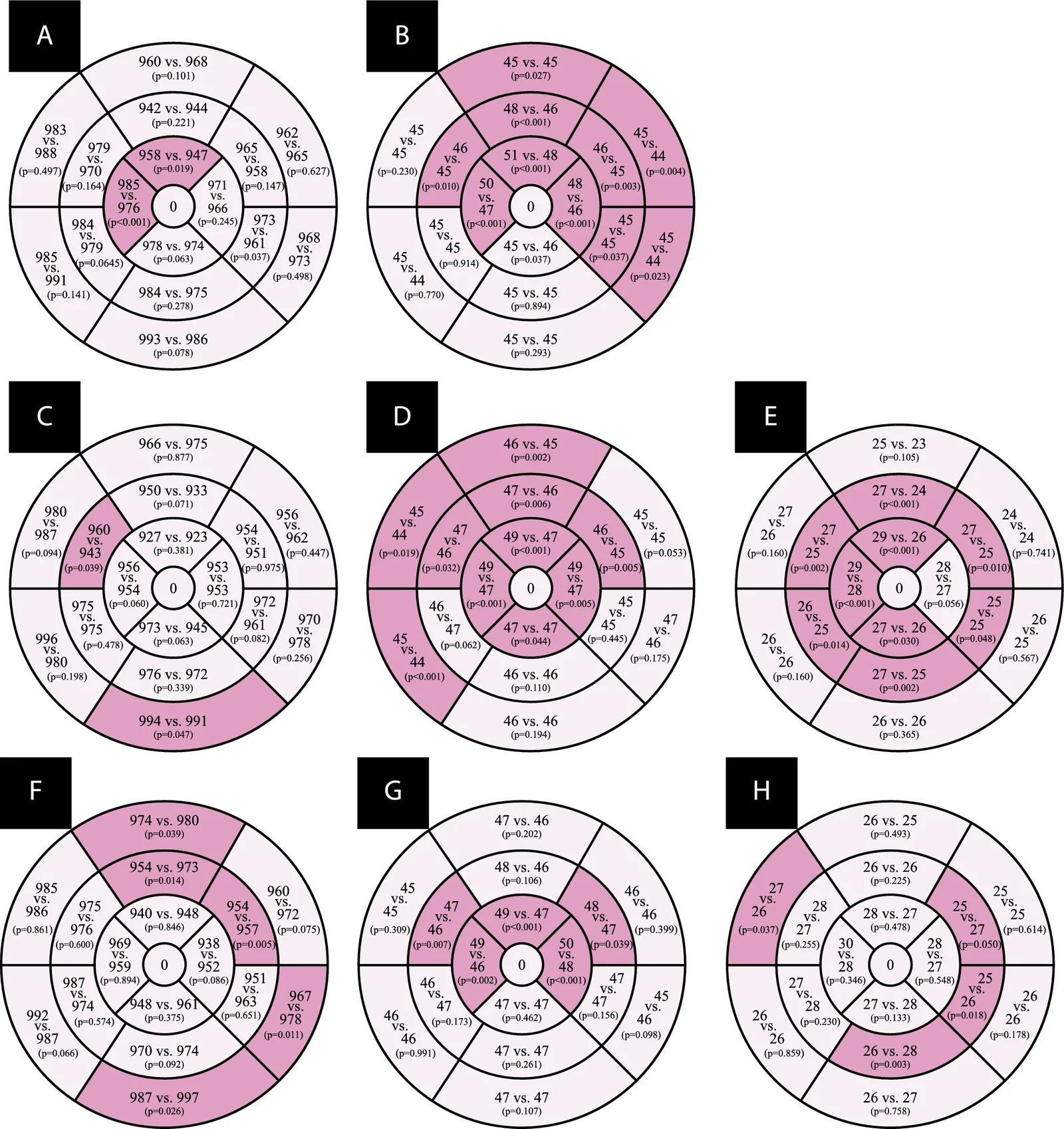
Diastolic and systolic segmental T1 and T2 relaxation times and extracellular volume (ECV) quantifications of all study cohorts displayed in the American Heart Association (AHA) Bull’s-eye plots. Panels **A, B** show T1 (A) and T2 (B) relaxation times for the healthy cohort. Panels **C–E** show T1 (left), T2 (middle), and extracellular volume (ECV, right) for the early rheumatoid arthritis (ERA) cohort (middle row). Panels **F–H** show T1 (left), T2 (middle), and ECV (right) for the aortic stenosis (AS) cohort (bottom row). T1 and T2 relaxation times are reported in milliseconds and ECV in percentages. Segments marked with pink color indicate a statistically significant difference (*p* value < 0.05) in mapping results

Systolic and diastolic phase segmental AHA Bull’s-eye plots of T1 and T2 relaxation times and ECV quantifications are provided in Fig. 3. In the segmental analysis, some segments showed variance in the mapping results between diastolic and systolic phase mapping, and these segments were scattered without any discernible pattern.

### Segments with artifacts

To assess image quality, segments with artifacts were categorized into two groups: minor artifacts and major artifacts leading to exclusion of the segment. The results are presented in Table 4.

**Table 4:** The frequency of minor and major imaging artifacts in each study cohort in diastolic phase and systolic phase mapping

	Diastolic phase		Systolic phase		*p* value^a^	*p* value^b^
	Minor artifacts	Major artifacts	Minor artifacts	Major artifacts		
Healthy cohort (*n* = 864 segments)						
T1 mapping, *n* (%)	121 (14)	21 (2)	46 (5)	4 (0.5)	< 0.001	0.015
T2 mapping, *n* (%)	47 (5)	8 (1)	13 (2)	4 (0.5)	< 0.001	0.461
ERA group (*n* = 480 segments)						
T1 mapping, *n* (%)	104 (22)	13 (3)	50 (10)	6 (1)	0.001	0.223
T2 mapping, *n* (%)	26 (5)	3 (1)	7 (1)	0 (0)	0.007	0.180
ECV, *n* (%)	110 (23)	28 (6)	34 (7)	6 (1)	< 0.001	0.006
AS group (*n* = 480 segments)						
T1 mapping, *n* (%)	65 (14)	9 (2)	56 (12)	16 (3)	0.518	0.263
T2 mapping, *n* (%)	56 (12)	7 (1)	12 (3)	1 (0.2)	0.004	0.109
ECV, *n* (%)	89 (19)	33 (7)	59 (12)	24 (5)	0.036	0.151

*ERA* early rheumatoid arthritis, *ECV* extracellular volume, *AS* aortic stenosis

^a^*p* value for the comparison of number of segments with minor artifacts in diastolic and systolic mapping

^b^*p* value for the comparison of segments with major artifacts between diastolic and systolic phase

In the healthy cohort, systolic phase mapping yielded significantly fewer minor artifacts for both T1 and T2 (14% vs 5%, *p* < 0.001 for T1; 5% vs 2%, *p* < 0.001 for T2). Similar findings were observed in the ERA group for both T1, T2, and ECV, as well as in the AS group for ECV and T2 mapping. While minor artifacts decreased during systolic T1 mapping in the AS group, this difference lacked statistical significance (14% vs 12% segments, *p* = 0.518).

Except for T1 mapping in AS group, all groups showed a reduction in the number of segments with major artifacts in the systolic phase mapping. However, this reduction mostly remained statistically insignificant. In the ERA group, the prevalence of major artifacts in ECV quantification significantly decreased in systolic phase mapping (6% vs 1%, *p* = 0.006). Similarly, in the healthy cohort, major artifacts in T1 significantly reduced between diastolic and systolic phase mapping (2% vs 0.5%, *p* = 0.015).

**Fig. 4:**
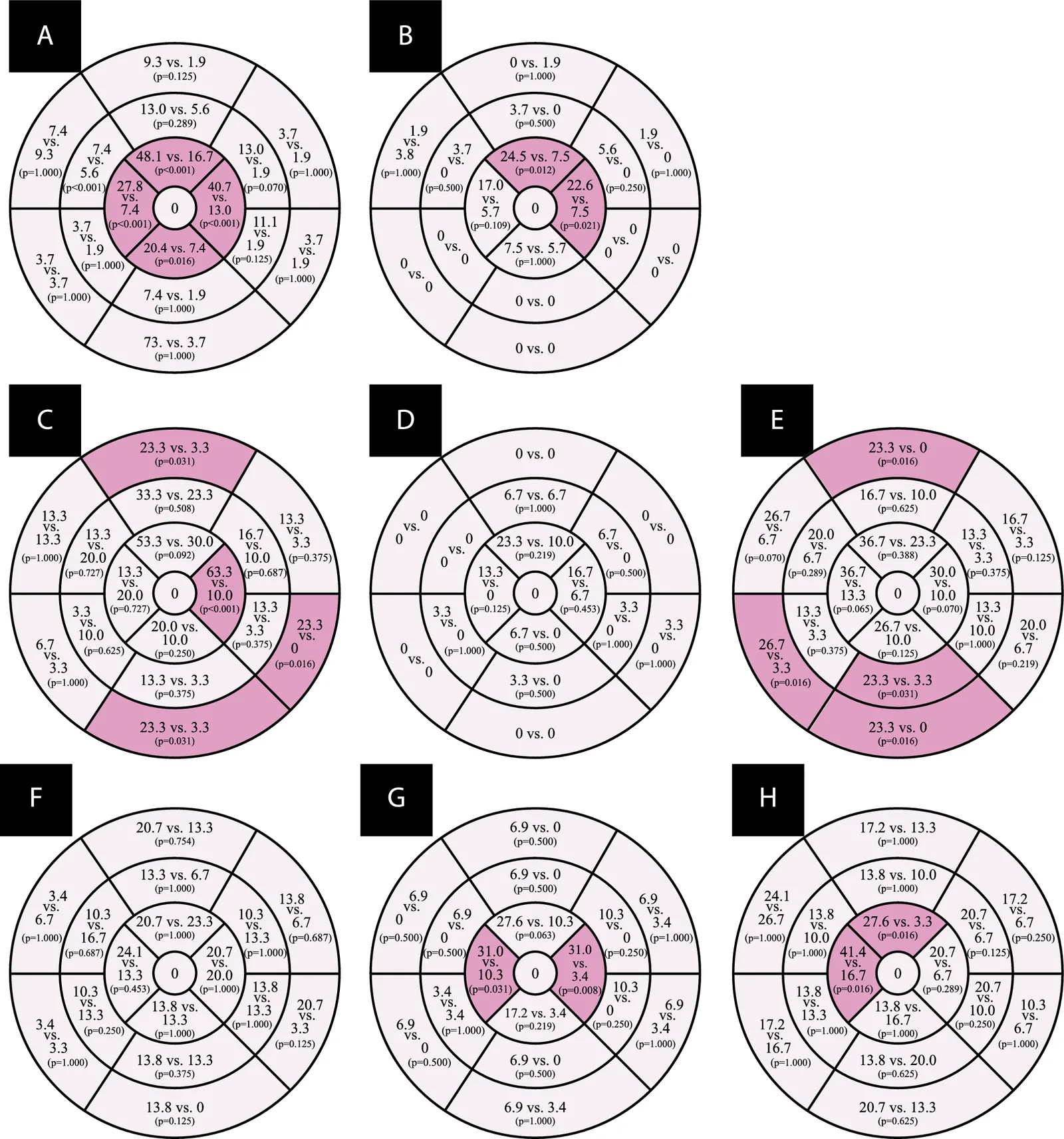
Segmental location and frequency of any artifacts in diastolic versus systolic phase segmental mapping, illustrated in American Heart Associations (AHA) Bull’s-eye plots. Images **A, B** show artifacts in T1 (left) and T2 (right) mapping in the healthy cohort. **C–E** and **F–H** display artifacts in T1 (left), T2 (middle), and extracellular volume mapping (right) for the early rheumatoid arthritis (middle row) and aortic stenosis cohorts (bottom row). The values are shown as percentages of segments affected by artifacts in the diastolic versus systolic phase mapping. The highlighted segments indicate a statistically significant difference in the frequency of artifacts between systolic and diastolic mapping

Additionally, we analyzed the distribution of artifacts within the myocardium using the AHA Bull’s-eye plots (Fig. 4). In diastolic and systolic phase T1 and T2 mapping, most artifacts were predominantly observed in the apical segments, with no consistent pattern of distribution noted elsewhere. In ECV, artifacts divided more widely, and a larger number of segments with a high frequency of artifacts (20% or more) were observed. The segments with the most artifacts partially overlapped with those segments showing significantly different mapping values between diastolic and systolic phase mapping (Fig. 3).

## Discussion

This study investigated T1 and T2 relaxation times and ECV quantification in diastolic and systolic mapping across three cohorts: a healthy control group, patients with ERA, and patients with AS. Our findings indicate that systolic mapping substantially improves image quality by reducing artifacts, while yielding comparable myocardial T1, T2 relaxation times, and ECV values to diastolic mapping, across diverse patient profiles with varying myocardial thicknesses. Systolic mapping also improves the repeatability of myocardial mapping, supported by the higher ICC values.

We classified artifacts as either minor or major, with major artifacts rendering segments unreadable. Our study demonstrated that systolic phase mapping significantly reduces the number of segments with minor artifacts compared to the gold-standard diastolic mapping. This finding supports the growing body of evidence, suggesting that systolic mapping enhances image quality [18, 20, 21]. The reduction in minor artifacts in mapping results is notable, since those artifacts would normally otherwise impact the overall mapping values.

Although the number of segments with major artifacts decreased between diastolic and systolic phase mapping, this reduction was mostly not statistically significant. The reduction in minor artifacts was most pronounced in cohorts with normal or thin myocardial walls, highlighting the advantage of systolic mapping for these groups. However, in the AS cohort with thicker myocardial walls, systolic mapping offered only slight artifact reduction, suggesting limited additional benefit for these patients. In fact, systolic ECV analyses may even be more difficult in patients with an extremely hypertrophic myocardium because of the limited visibility of free blood pool needed for normalization of the values with hematocrit.

In this study, imaging artifacts in both diastolic and systolic T1 and T2 mapping were mostly located in the apical segments (Fig. 4). In ECV mapping, a greater number of segments with > 20% artifacts were observed, and these artifacts distributed more evenly throughout the myocardium. Segments with the highest artifact frequency partially overlapped with those showing significant differences in T1, T2, and ECV values between diastolic and systolic mapping. However, some segments with variable T1 and T2 relaxation times had fewer artifacts, while other segments with artifacts showed no significant differences between diastolic and systolic mapping. These findings emphasize the need for further research into artifact distribution and impact. Notably, segments with major artifacts were classified as unreadable and excluded from comparisons.

Mapping sequences are artifact-sensitive due to factors, such as ECG mistriggering, inadequate magnetization recovery, or challenges in maintaining a breath-hold [18]. Systolic mapping can minimize these issues while providing comparable T1 relaxation times between phases [20]. Our study adds to previous research by standardizing systolic mapping across a broader population, including healthy adult volunteers and clinical patients with varied myocardial thickness profiles. The ERA cohort represented thinner myocardial walls, while the AS cohort included ventricular hypertrophy. In our study, T1 and T2 relaxation times were consistent between diastolic and systolic mapping across all participant profiles. Notably, our healthy cohort of 54 adults, balanced by gender (50% female, 50% male) and spanning a wide age range (18–65 years), provides a robust reference for mapping in a healthy population.

Systolic mapping may provide practical advantages related to cardiac timing. Systolic quiescence is generally more stable across varying heart rates than diastolic quiescence, particularly in patients with higher or irregular heart rates, where diastolic duration shortens and becomes more variable. This relative stability may simplify trigger-delay prescription during clinical scans and reduce sensitivity to heart-rate-dependent timing errors, especially during breath-hold acquisitions. Although the present study was not designed to specifically assess heart-rate variability or trigger robustness, the improved repeatability observed with systolic mapping may in part reflect more consistent cardiac timing. It should be noted that systolic triggering may slightly limit the achievable inversion time range in MOLLI-based acquisitions; however, this is unlikely to be clinically relevant for myocardial T1 values at 1.5 T. Furthermore, the acquisition window of the mapping sequence may extend over systolic phase. This may introduce additional blurring of the edges in the image, while the primary contrast signal still reflects the systolic phase.

Previous studies on systolic mapping in healthy subjects report variable T1 relaxation times between cardiac phases, largely attributed to partial-volume effects and reduced intramyocardial blood pooling, which affect quantification accuracy [18, 27, 28]. In the present study, improved image quality and repeatability with systolic mapping primarily reflect reduced susceptibility to motion-related artifacts and more stable myocardial boundary delineation, rather than elimination of these known confounding factors. Partial volume effects, myocardial thickness, intramyocardial blood pool contribution, and ROI location remain relevant determinants of absolute T1 values in both cardiac phases.

By selecting a mid-ventricular septal ROI, our study minimized known sources of variability and improved repeatability compared with global or segmental mapping, particularly in artifact-prone cohorts [5, 8, 17]. However, mid-septal ROI may not capture myocardial pathologies affecting specific areas of the myocardium, where segmental mapping remains essential [23]. Overall, our findings support the previous research demonstrating that targeted systolic mapping can refine measurement accuracy across cohorts with differing myocardial characteristics, while acknowledging persistent physiological and geometrical confounders [29].

The ERA cohort demonstrated a slight but significant reduction in ECV during systolic phase mapping, likely due to partial-volume effects and reduced intramyocardial blood volume contamination [18, 19, 30]. Rheumatoid arthritis patients often exhibit thinner myocardial walls and reduced left-ventricular mass [25]. Consistently, our ERA group showed a smaller left-ventricular mass compared to healthy cohort (85.6 g vs 97.3 g, *p* = 0.047). While systolic mapping mitigates partial-volume effects by increasing myocardial wall thickness, the thinner myocardial walls in the ERA group likely explain the ECV differences. In contrast, ECV values were consistent between phases in the AS cohort, which has thicker myocardial walls.

Systolic phase mapping yielded consistently higher ICC values when compared to diastolic phase mapping, particularly for T2 and ECV mapping. This suggests that systolic mapping provides slightly more consistent results, likely due to less-sensitive myocardial boundary delineation during systole, where thickened myocardium reduces variability. The result is an important observation in terms of measurement reproducibility directly affecting the sensitivity of the follow-up studies. Ultimately, this may provide tools to detect smaller changes in the heart viability and enable more timely response on the changes.

## Limitations

This study has limitations. Although our cohorts were relatively large, sample size limits generalizability. The CMR protocol employed ShMOLLI sequences, and the results are, therefore, specific for this imaging technique. While slice positioning errors are not artifacts as such, they occur in clinical workflows and affect the interpretation and reproducibility of the mapping results similarly to motion artifacts. Thus, they were included in our analyses, and some artifacts, especially in segment 6, may partially reflect suboptimal slice positioning rather than technical artifacts.

The artifact categorization was based on separate analyses by one cardiac radiologist, not clinical reader assessment. Thus, intra- and inter-reader variability of the artifact categorization was not studied. Appearance of the artifacts may also depend on the applied relaxation map calculation method and may be difficult to differentiate from clinical findings. In our image acquisition and analysis workflow, artifacts were mostly either clearly motion-induced, typical banding or single abnormal pixels resulting from poor relaxation model fitting. Misinterpretation cannot, however, be fully excluded.

Patients with arrhythmias or hypertrophic cardiomyopathies (HCM) were excluded. Tachyarrhythmias can pose challenges for systolic mapping due to ECG mistriggering at higher heart rates. Arrhythmias may, however, affect the quality of diastolic mapping as well, and in some circumstances, it might be useful to perform mapping both in diastolic and systolic phases. In HCM, the location of the hypertrophic myocardium can affect systolic mapping values, particularly in apical HCM, where diastolic phase mapping could facilitate the blood pool contouring in ECV quantification. Future studies with larger cohorts and varied mapping approaches are needed to validate these findings.

## Conclusion

This study assessed T1, T2, and ECV values in diastolic and systolic phase mapping across healthy individuals, ERA, and AS patients. Systolic phase mapping significantly reduced imaging artifacts and improved mapping reproducibility, while providing comparable T1 and T2 relaxation times and ECV quantification to diastolic mapping, especially in individuals with normal or thin myocardial walls. This improvement in image quality, along with an excellent inter-observer agreement, suggests that systolic mapping may offer a more reliable alternative for gold-standard diastolic mapping, particularly in populations prone to imaging artifacts. Future research should further explore its benefits across diverse patient groups.

## Supplementary Information

Below is the link to the electronic supplementary material.

Supplementary file1 (PPTX 632 KB)

## Data Availability

The data underlying this article will be shared on reasonable request to the corresponding author.
